# The Effects of a Mediterranean Diet Intervention on Cancer-Related Fatigue for Patients Undergoing Chemotherapy: A Pilot Randomized Controlled Trial

**DOI:** 10.3390/cancers14174202

**Published:** 2022-08-30

**Authors:** Amber S. Kleckner, Jennifer E. Reschke, Ian R. Kleckner, Allison Magnuson, Andrea M. Amitrano, Eva Culakova, Michelle Shayne, Colleen S. Netherby-Winslow, Susan Czap, Michelle C. Janelsins, Karen M. Mustian, Luke J. Peppone

**Affiliations:** 1 Department of Pain and Translational Symptom Science, University of Maryland School of Nursing, Baltimore, MD 21201, USA; 2 University of Maryland Greenebaum Comprehensive Cancer Center, Baltimore, MD 21201, USA; 3 Division of Supportive Care in Cancer, Department of Surgery, University of Rochester Medical Center, Rochester, NY 14642, USA; 4 Wilmot Cancer Institute, Rochester, NY 14642, USA; 5 Department of Medicine, University of Rochester Medical Center, Rochester, NY 14642, USA; 6 Department of Pathology, University of Rochester Medical Center, Rochester, NY 14642, USA

**Keywords:** oncology, nutrition, supportive care, integrative oncology, metabolism, mitochondria

## Abstract

**Simple Summary:**

Cancer-related fatigue affects the majority of people undergoing chemotherapy for cancer. The Mediterranean Diet provides healthy macro- and micronutrients that promote energy production and counter known mechanisms that contribute to fatigue. Thus, we designed a Mediterranean Diet program specifically for patients undergoing chemotherapy that included food provision, education, a cookbook, and weekly telephone check-ins. In a two-arm randomized controlled trial (*n* = 33), we found that our program was safe and feasible; there was excellent adherence (>70%) to the Mediterranean Diet. The Mediterranean Diet program led to less fatigue to a small-moderate degree at weeks 4 and 8. For those with a lower Mediterranean Diet score before the program, the program had a larger effect. Mitochondria are the cellular organelles that produce ATP energy and, in circulating T cells, fatigue was associated with mitochondrial dysfunction. These data support larger studies testing *how* and *how much* a Mediterranean Diet during chemotherapy can alleviate fatigue.

**Abstract:**

Cancer-related fatigue is a common, burdensome symptom of cancer and a side-effect of chemotherapy. While a Mediterranean Diet (MedDiet) promotes energy metabolism and overall health, its effects on cancer-related fatigue remain unknown. In a randomized controlled trial, we evaluated a rigorous MedDiet intervention for feasibility and safety as well as preliminary effects on cancer-related fatigue and metabolism compared to usual care. Participants had stage I–III cancer and at least six weeks of chemotherapy scheduled. After baseline assessments, randomization occurred 2:1, MedDiet:usual care. Measures were collected at baseline, week 4, and week 8 including MedDiet adherence (score 0–14), dietary intake, and blood-based metabolic measures. Mitochondrial respiration from freshly isolated T cells was measured at baseline and four weeks. Participants (*n* = 33) were 51.0 ± 14.6 years old, 94% were female, and 91% were being treated for breast cancer. The study was feasible, with 100% completing the study and >70% increasing their MedDiet adherence at four and eight weeks compared to baseline. Overall, the MedDiet intervention vs. usual care had a small-moderate effect on change in fatigue at weeks 4 and 8 (ES = 0.31, 0.25, respectively). For those with a baseline MedDiet score <5 (*n* = 21), the MedDiet intervention had a moderate-large effect of 0.67 and 0.48 at weeks 4 and 8, respectively. The MedDiet did not affect blood-based lipids, though it had a beneficial effect on fructosamine (ES = −0.55). Fatigue was associated with mitochondrial dysfunction including lower basal respiration, maximal respiration, and spare capacity (*p* < 0.05 for FACIT-F fatigue subscale and BFI, usual fatigue). In conclusion, the MedDiet was feasible and attenuated cancer-related fatigue among patients undergoing chemotherapy, especially those with lower MedDiet scores at baseline.

## 1. Introduction

Cancer-related fatigue describes tiredness that is experienced upon the development of cancer, after diagnosis, into treatment, and beyond [1]. It is nearly universal, affecting approximately 90% of patients undergoing chemotherapy [1,2,3]. It is not related to recent activity or relieved by rest, and its severity can greatly hinder the ability to perform day-to-day activities and decrease quality of life [4]. The term cancer-related fatigue refers to fatigue that stems from the physical neoplasm, the treatment, and the emotional experience surrounding the cancer experience, as it is difficult to discern the extent to which each of these contributes to fatigue [1]. As such, the mechanisms underlying the predisposition of an individual to cancer-related fatigue, precipitation of fatigue to high severity, and persistence of fatigue into survivorship remain poorly understood [5,6]. Recent research shows cancer-related fatigue is related to heightened inflammation, excess oxidative stress, metabolic dysfunction, dysfunction of mitochondria and ATP metabolism, imbalances in the gut microbiome, psychosocial stress, physical deconditioning, hypothalamic-pituitary-adrenal axis dysfunction, and others [7,8]. Treatment can trigger, exacerbate, and/or perpetuate cancer-related fatigue by increasing the risk of anemia, exacerbating underlying depression, increasing inflammation, and/or damaging mitochondria [4,9]. However, the limited understanding of the etiology and pathophysiology of cancer-related fatigue has continued to thwart the development and optimization of effective prophylactics and treatments.

In theory, nutritional interventions could help reduce precipitation and persistence of cancer-related fatigue via attenuating chemotherapy-induced inflammation and metabolic dysfunction, correcting nutrient deficiencies, reducing oxidative stress, and conferring general health benefits [10,11,12]. Additionally, patients possess a strong desire for nutritional interventions, especially during chemotherapy—patients report a sense of empowerment by using diet and nutrition to directly help themselves heal [13], leading to psychological improvements that may reduce fatigue. In fact, at least 43% of patients take nutritional supplements during treatment, despite evidence that some supplements (e.g., antioxidants) are associated with an increased risk of recurrence and poorer survival [14]. Moreover, “oral complementary and alternative medicine,” defined as taking homeopathy, vitamins/minerals, herbal supplements, or other dietary supplements, was associated with worse cancer-related fatigue during cancer treatment [15]. Other pleiotropic behavioral interventions such as exercise and cognitive behavioral therapy are the most effective interventions available to combat cancer-related fatigue and are more effective than available pharmaceutical interventions [16]. Nevertheless, fewer than a dozen studies have looked at nutrition therapy to address cancer-related fatigue during or after treatment, testing interventions such as Weight Watchers, Nordic nutrition guidelines, and a custom Fatigue Reduction Diet [10,11,17]. Due to the diversity of the interventions, there is currently insufficient evidence to recommend specific dietary patterns, but preliminary evidence suggests that a plant-based dietary pattern may alleviate fatigue [11].

The Mediterranean Diet (MedDiet) is a well-known health-promoting dietary pattern based on the traditional diets in the Mediterranean region. It includes high consumption of fruit, vegetables, legumes, nuts, seeds, whole grains, and olive oil; moderate consumption of seafood, dairy products (i.e., cheese and yogurt), eggs, poultry, and red wine with meals; and low consumption of sweets, red meat, and highly processed foods. The MedDiet has biological plausibility to address fatigue because it contains nutrients that are anti-inflammatory, anti-oxidative, and particularly beneficial for metabolism including protein, dietary fiber, polyphenols, and hydroxytyrosol [18,19,20]. It has been shown to be correlated with less severe fatigue in other conditions (e.g., multiple sclerosis [21], type 2 diabetes [22], myalgic encephalomyelitis/chronic fatigue syndrome [23]). Furthermore, MedDiet promotes the health of the gut microbiome [24], the balance between pro- and anti-inflammatory responses [25,26], sleep quality [27], and improvements in mood and depressive symptoms [28,29]. This diet is appealing, too, because it is accessible, adaptable, highly palatable, and not overly restrictive.

While MedDiet promotes general health and has biological plausibility to prevent fatigue in clinical research, it has not yet been tested in a randomized controlled trial during chemotherapy treatment to address fatigue. Additionally, it is unknown how a MedDiet intervention can affect metabolic biomarkers and mitochondrial function in the context of chemotherapy treatment. Thus, we evaluated the feasibility of delivering an 8-week MedDiet intervention during chemotherapy treatment for cancer and the preliminary efficacy of the MedDiet intervention on cancer-related fatigue. We hypothesized that the MedDiet intervention would be feasible and would lead to less fatigue at four and eight weeks, improved blood-based metabolic markers, and improved mitochondrial function, compared to a usual care control condition.

## 2. Materials and Methods

### 2.1. Study Design

The Diet and Nutrition in Cancer (DANICA) trial was a pilot randomized controlled trial conducted at the Wilmot Cancer Institute (WCI) from November 2020 to December 2021. This study was preregistered at clinicaltrials.gov (identifier: NCT04534738, registered September 2020). The research protocol was reviewed and approved by the Institutional Review Board. The primary aims of the trial were to evaluate the feasibility of delivering a MedDiet intervention, as measured by adherence at both four weeks and eight weeks, and to evaluate the preliminary efficacy of MedDiet vs. usual care to prevent cancer-related fatigue among patients with cancer undergoing chemotherapy. Secondary aims were to evaluate the effects of the MedDiet on blood-based metabolic biomarkers (e.g., fructosamine) and ex vivo mitochondrial function (i.e., mitochondrial stress test of T cells).

### 2.2. Participants

Individuals were eligible for this study if they had a diagnosis of stage I–III cancer (solid tumor or hematological malignancy), were scheduled to receive chemotherapy and had at least six weeks remaining, could communicate in English, were at least 18 years old, were willing to adhere to study procedures, and were able to provide written informed consent. Individuals were not eligible if they were on enteral or parenteral nutrition, were pregnant, had a brain tumor, were planning to get radiation to the head, had specific dietary needs that a MedDiet could not meet, or were already following the MedDiet (i.e., have a score ≥ 10 on a modified 14-item MedDiet questionnaire [30]). (Exclusion of those with a brain tumor or plans to get radiation to the head was recommended by a registered dietitian at WCI because these patients tend to have frequent and extreme changes in taste and food preferences.) In addition to patients who ate meat, patients who were following a vegetarian or vegan diet were eligible because it is possible to adhere to the MedDiet without eating meat or animal products.

### 2.3. Procedures

Patients were referred to study investigators by clinicians at WCI or responded to flyers advertising the study. Volunteers interested in participating completed a telephone screening to verify eligibility. Participants were provided the choice to consent via a paper consent or eConsent using REDCap software [31,32]. For baseline assessments, participants completed questionnaires, a three-day food record, and a blood draw (described in detail in Section 2.6). Participants were then randomized 2:1, intervention:control, using a computer-generated random numbers table with blocks of 3 or 6 and stratified based on whether their chemotherapy regimen was (1) weekly, biweekly, or every four weeks, or (2) other; stratification occurred to ensure that participants were balanced in regard to whether baseline, 4-week, and 8-week measurements were at the same vs. different times in their chemotherapy cycle. The same assessments were administered in week 4 and week 8. Participants in both groups were called weekly during the 8-week study by the same researcher (AK) to promote engagement, assess adverse events, and if in the MedDiet group, troubleshoot lack of adherence.

### 2.4. MedDiet Intervention

The MedDiet intervention was eight weeks long. Upon randomization to the intervention arm, participants received a Mediterranean cookbook developed specifically for this program, 16 × 1-ounce packages of walnuts donated graciously by the California Walnut Commission, a 3-cup/750-mL glass rectangular container for reheating frozen meals, and an information packet describing the MedDiet including the MedDiet Pyramid (Fundación Dieta Mediterránea [33]) and the Mediterranean Exchange List [34]. While we asked about the wine consumption, we did not encourage alcohol consumption during the study. For the first four weeks, food compliant with the MedDiet was provided via weekly home delivery. This included 12 frozen meals per week from Project Lean Nation (Brighton, NY, USA), such as the Falafel Plate, Pan-Seared Salmon, and Baked Ziti from the “Lifestyle Plans” line. These meals were complemented with MedDiet staples from Wegmans (Rochester, NY, USA; e.g., low sodium V8 vegetable juice, olive oil, spinach, whole wheat pasta) and delivered via Instacart. In week 3, participants completed a one-on-one educational session with a nutrition scientist (AK) to promote behavior change, which was based on a combination of behavioral theory and cognitive behavioral theory [35]. This session was via phone or in-person and was 20–60 min long. Participants discussed strategies for behavior change including goal setting (e.g., three servings of vegetables per day), self-monitoring (e.g., keep a food log), and stimulus control (e.g., make sweets less accessible), which were listed in the information packet; participants recorded their action items. 

### 2.5. Control Arm

Those assigned to the control arm were neither encouraged nor discouraged to follow any particular diet. At the end of the study, participants in the control arm received the cookbook, walnuts, and one week’s worth of food from Project Lean Nation and Wegmans.

### 2.6. Measures

Questionnaires were administered at baseline, week 4, and week 8. Adherence was assessed using the 14-item Mediterranean Diet Assessment Tool, which has been used in epidemiological studies and clinical trials [30]. Scores range from 0–14, with a higher score indicating greater adherence. It does not specify a time period. Fatigue was assessed using the Functional Assessment of Chronic Illness Therapy-Fatigue (FACIT-F) [36], Brief Fatigue Inventory (BFI) [37], and the Symptom Inventory. The FACIT-F is a 40-item, validated patient-reported fatigue measure that is comprised of five subscales: physical well-being, social well-being, emotional well-being, functional well-being, and fatigue [36]. It asks how true various statements were over the last seven days such as “I have lack of energy” and “I have trouble starting things because I am tired” with five response choices ranging from 0, “Not at all,” to 4, “Very much.” Scoring requires reversing some responses to yield five subscale scores, a total score, a general quality of life score (Functional Assessment of Cancer Therapy-General [FACT-G]; physical + social + emotional + functional well-being), and a trial outcome index (TOI; physical well-being + functional well-being + fatigue subscale). A higher score indicates higher well-being/quality of life or less fatigue. The BFI is a 10-item fatigue questionnaire that is also validated and commonly used in oncology [37]. It captures fatigue *now* as well as the *usual* and *worst* fatigue in the last 24 hours from 0, “No fatigue”, to 10, “As bad as you can imagine”. It also includes six single-item questions regarding how fatigue has interfered with general activity, mood, etc., from 0, “Does not interfere”, to 10, “Completely interferes”. The average of all 10 items yields a global fatigue score with a higher score indicating worse fatigue. Cronbach alpha reliability ranges from 0.82 to 0.97 [37]. Lastly, we administered three questions related to fatigue as part of a Symptom Inventory—fatigue, sleep problems, and drowsiness—in addition to how much symptoms interfered with the quality of life. We asked the participants to rate the symptoms at their worst in the past seven days from 0, “Not present”, to 10, “As bad as you can imagine”.

Safety was measured by recording all adverse events Grade 3 or higher according to the Common Terminology Criteria for Adverse Events (CTCAE), version 5 [38], and attributing them to the intervention or not. A Grade 3 adverse event was defined as severe or medically significant but not immediately life-threatening; hospitalization or prolongation of hospitalization indicated; disabling; and/or limiting self-care activities of daily living. We paid special attention to weight loss and extracted body weight from the medical record at baseline, four weeks, and eight weeks. Body mass index (BMI) was calculated as body weight in kg/(height in m)^2^.

Three-Day food records were completed by participants at baseline, four weeks, and eight weeks. Data were entered into Nutrition Data System for Research (NDSR) software (2021 version; Nutrition Coordinating Center, University of Minnesota, Minneapolis, MN, USA) by a team of two trained registered dietitians (RP and NW). Nutrients from food and supplements were summed to estimate total dietary intake for macro- and micronutrients. The number of servings in each food group was estimated using NDSR’s Serving Count Totals analysis.

Blood was collected from either the participant’s implanted chemotherapy port or venipuncture at baseline, week 4, and week 8 into red-top tubes with no anticoagulant or preservative (for serum) and mint green-top tubes with sodium heparin (for mitochondrial assays). All tubes were inverted several times before processing. 

For serum processing, tubes sat upright at room temperature for 30 min to allow for clotting, then were centrifuged for 15 min at 1600× *g* at 4 °C. Serum was aliquoted into 2-mL microcentrifuge tubes and stored at −80 °C. Fructosamine was quantified by Associated Regional and University Pathologists, Inc. (Salt Lake City, UT, USA). Other metabolic assays were performed by URLabs (West Henrietta, NY, USA) and included homocysteine and a lipid panel (total cholesterol, triglycerides, and high-density lipoprotein [HDL]). Low-density lipoprotein (LDL) was calculated as (total cholesterol − HDL cholesterol − triglycerides)/5, and non-HDL cholesterol (NHDLC) was calculated as (total cholesterol − HDL). T cells were extracted from fresh blood, and cellular respiration was measured ex vivo on the Seahorse XFe96 Extracellular Flux Analyzer (Agilent Technologies, Santa Clara, CA, USA). Please see Appendix A for details.

### 2.7. Sample Size Determination and Statistical Analysis

Our sample size was determined *a priori* based on other studies of plausibility and considerations for pilot studies (e.g., [39,40]). The statistical power was addressed with respect to the FACIT-F fatigue subscale as our primary patient-reported outcome [41]. Assuming a correlation of 0.50 between FACIT-F fatigue measurements at baseline and week 4, evaluable data from *n* = 33 would provide 80% power at the 0.15 two-sided significance level to detect an effect size (ES, standardized mean between-group difference) of 0.75 of the intervention effect on change in FACIT-F fatigue using analysis of covariance (ANCOVA) with the FACIT-F fatigue baseline measure as a covariate. We factored in a 20−25% attrition rate, and our protocol was approved to recruit up to 42 participants; our stopping rule was *n* = 33 participants to provide post-intervention data. No interim analyses were planned or performed. 

Differences between groups at baseline were identified using a *t*-test for continuous variables and a χ^2^ test for categorical variables. To assess adherence, changes in the MedDiet Adherence questionnaire from baseline to week 4 and week 8 were compared using a two-sided *t*-test. For the effects of the MedDiet intervention on fatigue, food intake, and metabolic measures, as part of an intent-to-treat analysis, a mixed effect model was constructed with the measure at week 4 or 8 as the dependent variable; group, time, and group×time as fixed effects; the measure at baseline as an independent covariate; participant as a random effect; and an autoregressive structure (AR[1]) accounting for repeated measures (4, 8 weeks) per participant (JMP Pro 16, SAS Institute, Cary, NC, USA). The between-group difference (usual care vs. MedDiet) in the change in outcome (e.g., fatigue) was estimated via marginal means. To assess associations between mitochondrial function and patient-reported fatigue, mixed effects models were developed with fatigue as the dependent variable, the mitochondrial outcome and age as independent variables, participant as a random effect, and an AR(1) correlation structure. Effect sizes were calculated as Cohen’s *d* (ΔMedDiet − ΔControl)/SD_pooled_, where Δ is the change from baseline to week 4 or week 8 and SD_pooled_ is the standard deviation from all participants at baseline. The confidence interval (CI) of the effect size was calculated as described in Lee et al. [42]. A two-sided probability of *p* < 0.05 was considered statistically significant and, to avoid Type II errors, *p* ≤ 0.15 was considered meaningful for informing future research.

## 3. Results

### 3.1. Population

**Figure 1:**
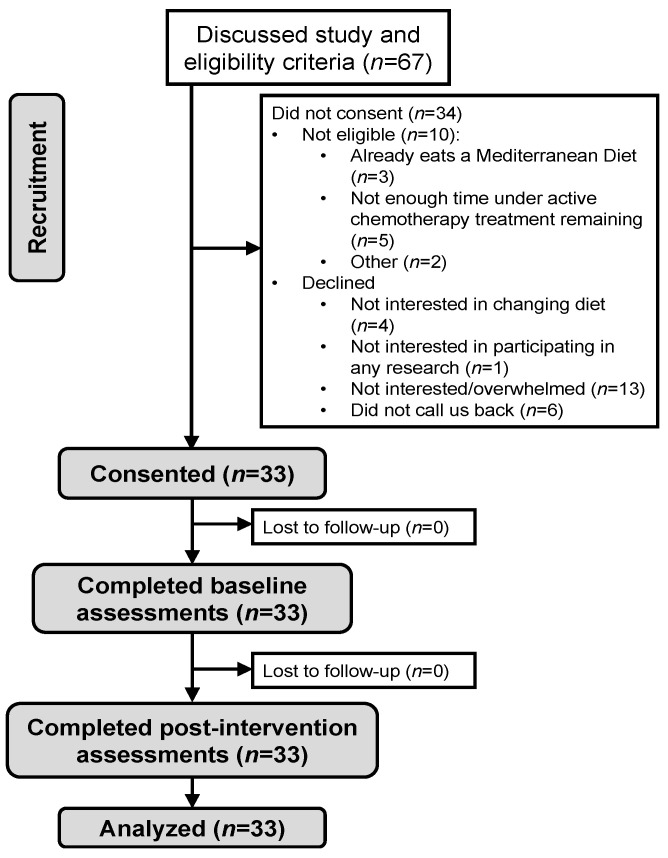
CONSORT diagram.

A total of 33 patients consented (Figure 1). Participants were 51.0 ± 14.6 (mean ± standard deviation (SD)) years old, the majority female (94%), Non-Hispanic White (88%), well-educated, and were being treated for breast cancer (91%) (Table 1). The majority (25/33, 75.8%) were overweight or obese, as defined by a BMI ≥ 25 kg/m^2^.

### 3.2. Feasibility, Dietary Adherence, and Safety

The threshold for study feasibility was set a priori at >70% of participants who consented to complete the study; 100% completed the study and provided fully evaluable data for the primary aim of fatigue. 

MedDiet adherence was measured using the 14-item Mediterranean Diet Assessment Tool. Scores were similar between groups at baseline (MedDiet 4.0 ± 2.7, control 4.7 ± 1.9, Table S1; *p* = 0.46). At week 4, scores increased from baseline by 2.7 ± 3.2 (mean ± SD) points for the MedDiet group and 0.7 ± 2.4 points for the control group (*p* < 0.001 and *p* = 0.44 within-group comparisons, respectively; *p* = 0.062, two-sided between-group comparison). Increases from baseline to week 8 were 2.5 ± 3.0 points for the MedDiet group and 0.5 ± 2.5 points for the control group (*p* < 0.001 and *p* = 0.54 within-group comparisons, respectively; *p* = 0.058, between-group comparison). We stated a priori that feasibility would be declared if >70% of participants in the intervention arm improved their MedDiet score at least one point from pre- to post-intervention—17/23 (74%) had improved at week 4 and 18/23 (78%) had improved at week 8. Thus, we confirmed feasibility.

Three-day food records were collected and analyzable from 25 participants (31 of 99 days were missing or unevaluable). There were no statistical differences between groups at baseline for intake of total energy, total fat, carbohydrates, protein, or fiber (*p* > 0.05; Table S2). On average, per week, those in the MedDiet group consumed eight more servings of vegetables, five more servings of whole grains, two more servings of beans/legumes, four more servings of nuts, 10 more servings of oil, and nine fewer servings of butter at week 4, with most differences maintained at week 8. However, with the large variation in both groups, only whole grains reached statistical significance (Table S2). Those in the MedDiet group reported at least 30% greater intakes of monounsaturated fats, zinc, and magnesium at week 8, which are high in olive oil and/or some nuts; statistical significance was only reached for magnesium. 

The MedDiet intervention was determined to be safe. A total of eight adverse events occurred—two were in the control group, five were in the MedDiet group, and one participant had consented but had not yet started the intervention. With two-thirds of participants in the MedDiet group, the distribution of events was relatively balanced. All adverse events were Grade 3 and not attributed to the intervention—shortness of breath, fainting after the first dose of chemotherapy, pneumonia, bacteremia, deep vein thrombosis, dental infection, neutropenic fever, and cellulitis of the chest wall. All participants who experienced adverse events continued the study.

### 3.3. Cancer-Related Fatigue

**Figure 2:**
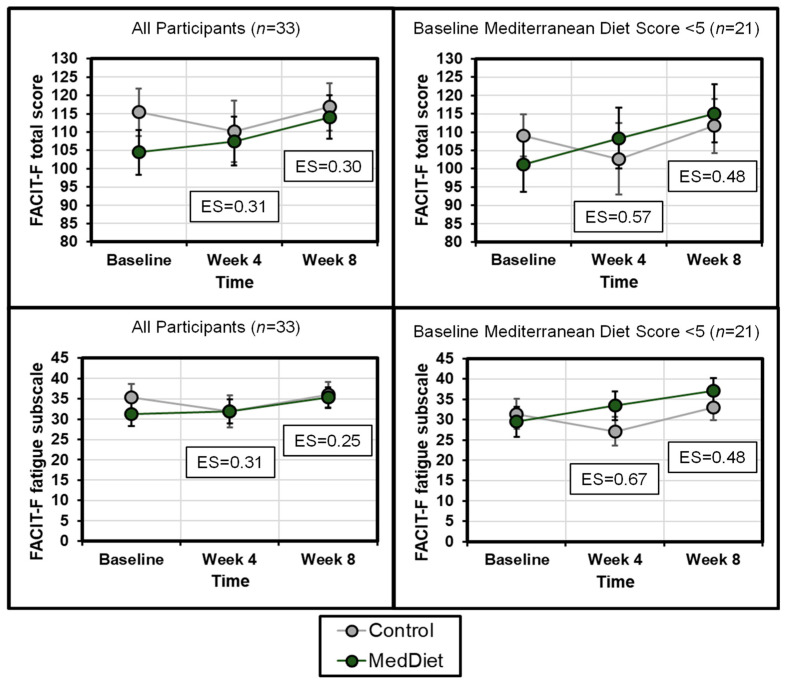
The Mediterranean Diet intervention was more effective for those with a lower Mediterranean Diet score at baseline. Cancer-related fatigue was measured using the Functional Assessment of Chronic Illness Therapy-Fatigue (FACIT-F) total score (top) and fatigue subscale (bottom). The left two panels show the effects of the intervention for all participants (*n* = 33), and the right two panels show the effects of the intervention among those with a baseline Mediterranean Diet Score < 5 on the Mediterranean Diet Assessment Tool (*n* = 21). For both the FACIT-F total score and FACIT-F fatigue subscale, a higher score indicates less fatigue. ES = effect size.

Fatigue did not change significantly over time for either group (Table 2). In an intent-to-treat analysis, the MedDiet intervention had small-moderate beneficial effect on fatigue at week 4 and week 8 (ES = 0.31 (95% CI = −0.44–1.06) and ES = 0.25 (95% CI = −0.50−0.99), respectively, for the FACIT-F fatigue subscale; Figure 2). In a secondary analysis, we next assessed how the MedDiet intervention affected fatigue for individuals who had low MedDiet scores at baseline. For the two-thirds of our cohort who had a baseline MedDiet score <5 (*n* = 21), the MedDiet intervention had moderate-large beneficial effect on change in fatigue on the FACIT-F fatigue subscale (ES = 0.67 (95% CI = −0.26–1.60) at week 4 and ES = 0.48 (95% CI = −0.44−1.40) at week 8) (Figure 2). Furthermore, for the FACIT-F total score, the MedDiet intervention had a moderate beneficial effect (ES = 0.57 (95% CI = −0.34–1.50) and ES = 0.48 (95% CI = −0.44–1.40)) at weeks 4 and 8, respectively).

Due to the large individual variability in individuals’ diets, we next assessed associations between MedDiet adherence and fatigue regardless of group assignment. At baseline, a higher MedDiet score was associated with less fatigue as measured using the FACIT-F fatigue subscale (b ± SE = 1.81 ± 0.87, *p* = 0.046), BFI global fatigue score (b = −0.38 ± 0.17, *p* = 0.031), and BFI ‘fatigue at its worst’ (b = −0.59 ± 0.21, *p* = 0.009), all adjusting for age (Table S3). To assess these associations over the course of the study, we constructed a repeated measures mixed model. Statistically significant associations were seen for the relationship between MedDiet adherence and FACIT-F fatigue subscale (*p* = 0.021), FACIT-F total score (*p* = 0.046), FACIT-F physical well-being (*p* = 0.035), FACIT-F TOI (*p* = 0.035), BFI global score (*p* = 0.006), BFI ‘usual fatigue’ (*p* = 0.023), BFI ‘worst fatigue’ (*p* = 0.003), and symptom interference with quality of life on the symptom inventory (*p* = 0.041; Table S4).

### 3.4. Metabolic and Mitochondrial Measures

Participants’ body weight and lipid profiles (i.e., cholesterol, triglycerides, HDL, LDL) were stable over the 8-week study (Figure S1). Furthermore, homocysteine, which is an indicator of vitamin B_12_ or folate deficiency [43], was stable over time and not different between groups. Fructosamine is a measure of average blood glucose over the prior 1–2 weeks [44]. Notably, at week 8, those in the MedDiet group had fructosamine levels 9.4 ± 4.9 µmol/L lower than those in the control group (ES (95% CI) = −0.55 (−0.94–−0.16); *p* = 0.067, controlling for baseline levels). 

To study mitochondrial function, we assessed real-time respiration from freshly isolated T cells from peripheral blood (Figure S2). There was no effect of the MedDiet on basal respiration (b ± SE = 0.147 ± 0.517, *p* = 0.780), maximal respiration (b ± SE = 0.373 ± 0.366, *p* = 0.324), or spare capacity (b ± SE = 0.517 ± 0.302, *p* = 0.107). We then assessed associations between mitochondrial function and patient-reported fatigue using a mixed model (independent of the intervention). Greater patient-reported fatigue was consistently associated with lower basal respiration, lower maximal respiration, and lower spare capacity, after adjusting for age (Table 3). Specifically, fatigue as measured using the FACIT-F fatigue subscale and the BFI (usual fatigue) was statistically significantly associated with lower basal respiration (*p* = 0.044 and *p* = 0.006), lower maximal respiratory capacity (*p* = 0.021 and *p* = 0.014), and lower spare capacity (*p* = 0.029 and *p* = 0.044).

## 4. Discussion

Herein, we evaluated the feasibility and effects of a MedDiet intervention on cancer-related fatigue, metabolic measures, and mitochondrial function during eight weeks of chemotherapy treatment. We observed that the MedDiet intervention was safe and feasible. Participants in the MedDiet arm displayed excellent adherence, with notable increases in servings of vegetables, whole grains, beans/legumes, nuts, and olive oil. The MedDiet intervention vs. usual care had a small-moderate beneficial effect on fatigue among all participants, and a moderate-large beneficial effect on fatigue for patients who had a low MedDiet score at baseline. In addition, we observed associations between MedDiet adherence and less fatigue at baseline and when incorporating all three time points. The 8-week MedDiet intervention led to lower fructosamine levels with a moderate effect size, which reflects lower average glucose concentration over the two weeks prior to blood sampling. Interestingly, we also observed associations between impaired mitochondrial respiration of circulating T cells and higher fatigue, which could be useful in understanding the mechanisms underlying fatigue, tailoring nutritional interventions to target mitochondrial function, as well as the development of objective biomarkers related to cancer-related fatigue.

Our data add to the growing body of literature that a MedDiet intervention is safe and feasible during active cancer treatment. Mediterranean-inspired diets have been conducted with success in other randomized controlled trials among patients undergoing chemotherapy including patients with lung cancer (inclusion criteria included patients on other active treatments as well) [45], breast cancer [46], and acute myeloid leukemia [47]. Gioxari et al. [45] administered a resource-intensive 3-month intervention with personalized nutritional counseling, daily diet plans, and educational booklets, delivered by experienced dietitians. To assess adherence, they used a different MedDiet score than used herein that was also based on MedDiet patterns including olive oil, fruits, vegetables, and fish (“Mediterranean Diet Score” with a range of 0–55 [48]). They observed a 5.5-point increase in score in the MedDiet group (*n* = 12) compared to a 1.5-point increase in the control group (*n* = 12, *p* = 0.031). Villarini et al. [46] provided individualized dietary recommendations and required cooking classes and common meals at least twice a week over the course of chemotherapy, with goals to prevent gastrointestinal side effects and reduce caloric intake. They measured compliance using six 24-hour recalls and reported differences between the intervention and control groups for whole grains and legumes (higher in the intervention group, *n* = 47), as well as white bread and refined grains, sugar, dairy, and processed meat (higher in the control group, *n* = 47) [46]. Jalali et al. [47] administered a 4-week personalized intervention under the direction of a nutritionist (*n* = 25 in the MedDiet group and *n* = 25 in the control group), however, they did not report adherence [47]. None of these studies reported fatigue. Our intervention was also intensive, with weekly food provision, weekly phone calls, educational handouts, and a custom cookbook. Future research should explore what elements of the dietary intervention—structured meal plans, individualized counseling, food provision, self-monitoring, social support, etc. [35]—are necessary and sufficient for successful MedDiet adoption, as well as the most economical.

Our findings that a nutritional intervention improves fatigue with a small-moderate effect are consistent with previous randomized controlled trials that have targeted cancer-related fatigue during cancer treatment. We observed an improvement in fatigue from baseline to week 8 of 0.7 points in the control group and 4.0 points in the MedDiet group; this difference in change score of 3.3 points is larger than the minimal clinically important difference (MCID) of 3 points [49]. Other studies investigating nutritional interventions to address fatigue include a 24-month nutritional support intervention for patients with gastrointestinal cancer (*n* = 70) [50], a “regular food” intervention during radiotherapy for patients with head and neck (*n* = 75) [51] and colorectal cancer (*n* = 111) [52], and a 6-month intensive nutrition intervention for patients with newly diagnosed esophageal or stomach cancer (*n* = 21) [53]. Collectively, three of four studies showed that the dietary intervention prevented fatigue [51,52,53]; Persson et al. [50] did not see an effect of the intervention on fatigue but did show a positive correlation between weight gain and fatigue. Our study adds to this literature that a MedDiet intervention, which was not specific to cancer type or individual clinical characteristics, may be promising to improve fatigue in the cancer treatment setting. Since this study was conducted only among patients undergoing active chemotherapy, future work is needed to assess whether the MedDiet intervention is effective to prevent chemotherapy-induced fatigue vs. addressing cancer-related fatigue that arises from other sources.

We observed that those with lower MedDiet scores at baseline had greater fatigue (Table S3) and experienced a greater effect of the intervention on their fatigue (Figure 2). This is consistent with previous literature that patients with lower adherence to health behaviors (i.e., diet, exercise) will experience the most benefit from the intervention [54]. We used an inclusion criterion cut-off of ≤9 points on a modified 14-point MedDiet Adherence scale herein. Our data suggest that a cut-off of ≤5 points may be more useful for inclusion criteria in future MedDiet studies and, potentially, the identification of individuals who will most benefit from a resource-intensive nutritional intervention. However, because this was a secondary analysis, these data are hypothesis-generating rather than hypothesis-testing.

Mitochondrial dysfunction is increasingly recognized as a contributor to fatigue due to mitochondria’s essential role in energy production, generation and regulation of reactive oxygen species, and other physiological processes [8,55]. Mitochondria are inadvertent targets of chemotherapy, especially doxorubicin and oxaliplatin [56]; chemotherapy causes damage to the mitochondrial structure and function, as well as its DNA. Further, mtDNA is more susceptible to mutations and has poorer repair mechanisms than nuclear DNA, likely predisposing it to worse damage from chemotherapy [57]. Herein, we showed that patient-reported fatigue was associated with lower overall mitochondrial function, namely lower basal respiration, lower maximal respiration, and a lower spare capacity in circulating T cells. There have been nine studies to our knowledge probing mitochondrial function in the context of cancer-related fatigue in various cell types [58,59,60,61,62,63,64,65]. Six of these studies looked at associations between mitochondrial function and fatigue during treatment from radiotherapy [58,59,60,61,63,64], one enrolled patients initiating anthracycline- or taxane-based chemotherapy [62], and one recruited breast cancer survivors who underwent a variety of antineoplastic treatments [65]. Collectively, these studies show that fatigue was associated with less mtDNA [62], downregulated mitochondrial gene expression [59,61], and less complex III-linked respiration [60,61], but no changes in the activity of mitochondrial oxidative phosphorylation complex enzymes [63], all in circulating blood cells. Our data are consistent with these findings and go beyond by demonstrating clear associations between ex vivo impairments in mitochondrial respiration and patient-reported fatigue.

Our mitochondrial data in isolated T cells are of particular interest because dysregulated T cell metabolism has been associated with impaired T cell function [66]. Chronic exposure to antigens, inflammation, and nutrient deprivation in cancer settings drives metabolic insufficiency and progressive loss of antitumor effector functions. These metabolic alterations drive T cells towards exhaustion, a state of hypo-responsiveness that has been observed in chronic infection and cancer. Future research should explore how the observed impairment in T cell respiration relates to T cell function and chronic inflammation.

The MedDiet is not currently in nutrition guidelines during chemotherapy treatment [67,68,69], though it is consistent with most of the recommendations. Patients should work with a registered dietitian specializing in oncology throughout their treatment and survivorship to manage symptoms, improve clinical outcomes, and prevent cancer recurrence. Older patients, in particular, may be more susceptible to malnutrition, cachexia, and poor dentition or denture issues [70], and may need more comprehensive intervention. Diets that focus on supportive care outcomes should also consider the effects on cancer progression and anti-neoplastic effects of chemotherapy; a systematic review and meta-analysis of observational studies showed that adherence to the MedDiet was associated with a significant reduction of risk for overall cancer mortality [71].

There are many strengths to this study. The program was conducted completely remotely using home-delivered food and telephone calls, thereby making it accessible to home-bound populations. While our frozen meal supplier was local and had a limited shipping radius, larger studies could utilize nationwide food delivery and shipping services. It was not tailored to the individual for calorie or macronutrient intake as in other studies (e.g., [46,47]), thereby reducing the resources of a registered dietitian. This study employed patient-reported outcomes and objective metabolic measures at the same time points, allowing us to directly probe underlying metabolic mechanisms of fatigue and how diet affected these measures.

This study, however, is not without limitations. Participants were mostly highly educated, non-Hispanic White women with breast cancer, so generalizability to other populations should be done with prudence. Furthermore, fatigue fluctuates over chemotherapy cycles and these data are only from three time points; therefore, these data did not capture a detailed trajectory of fatigue. However, we used multiple instruments that capture multidimensional aspects of fatigue over different time frames. We also stratified our participants based on chemotherapy cycle length so that the timing of our assessments in relation to their chemotherapy was balanced between groups. Our blood sampling was scheduled in conjunction with clinical appointments, and therefore was not necessarily fasted, which might have caused increased variability in the blood-based measures. However, random measurements are clinically useful and some measures such as fructosamine are not affected by fasting status [44]. This study was conducted during the coronavirus pandemic, which could have affected people’s adherence to a MedDiet intervention (e.g., more facilitators with less travel, socializing, and eating out; more barriers with higher stress and uncertainty, less social support). Participants were not blinded due to the nature of the intervention, and analysis was not blinded due to the high involvement of the Principal Investigator (AK) in intervention delivery and data analysis. Additionally, the usual care arm was not a time- and attention-control intervention, which could have led to expectation bias in the intervention group.

## 5. Conclusions

This was the first study, to our knowledge, that assessed the effects of MedDiet on cancer-related fatigue during chemotherapy. The diet was feasible and safe, and participants had high adherence. We saw small-moderate beneficial effects of the MedDiet on fatigue, especially for participants who had low MedDiet scores (<5) at baseline. In addition, we observed that patients with worse fatigue had a worse mitochondrial function, further elucidating the pathophysiology underlying this syndrome. This study supports further evaluation of MedDiet in the clinical setting to prevent cancer-related fatigue through more definitive phase II/III clinical trials.

## Figures and Tables

**Table 1:** Demographics and clinical characteristics.

Demographics and Clinical Characteristics	All Participants (*n* = 33)	MedDiet (*n* = 23)	Usual Care (*n* = 10)
**Age (years, mean ± SD)**	51.0 ± 14.6	51.7 ± 14.2	49.2 ± 16.3
**Gender, *n* (%)**			
Male	2 (6.1%)	2 (8.7%)	0
Female	31 (93.9%)	21 (91.3%)	10 (100%)
**Race and Ethnicity, *n* (%)**			
Asian	2 (6.1%)	2 (8.7%)	0
Black/African American	2 (6.1%)	1 (4.3%)	1 (10%)
Hispanic, any race	0	0	0
White, Non-Hispanic	29 (87.9%)	20 (87.0%)	9 (90%)
**Marital status, *n* (%)**			
Married or long-term significant other	25 (75.8%)	18 (78.3%)	7 (70%)
Divorced, separated, single, or widowed	8 (24.2%)	5 (21.7%)	3 (30%)
**Employment, *n* (%)**			
Employed (including self-employed)	20 (60.6%)	13 (56.5%)	7 (70%)
Homemaker, unemployed, or retired	13 (39.4%)	10 (43.5%)	3 (30%)
**Highest level of education, *n* (%)**			
Less than a high school degree	0	0	0
High school/GED	3 (9.1%)	1 (4.3%)	2 (20%)
2- or 4-year degree or some college	16 (48.5%)	11 (47.8%)	5 (50%)
Graduate degree	14 (42.4%)	11 (47.8%)	3 (30%)
**Body mass index (kg/m^2^, mean ± SD)**	29.4 ± 6.8	28.9 ± 6.1	30.5 ± 8.5
**Type of cancer, *n* (%)**			
Breast	30 (90.9%)	20 (87.0%)	10 (100%)
Other	3 (9.1%)	3 (13.0%)	0
**Cancer stage, *n* (%)**			
Stage 1	8 (24.2%)	4 (17.4%)	4 (40%)
Stage 2	21 (63.6%)	17 (73.9%)	4 (40%)
Stage 3	2 (6.1%)	1 (4.3%)	1 (10%)
Other or Unknown	2 (6.1%)	1 (4.3%)	1 (10%)
**Previous treatment for cancer, *n* (%)**			
Surgery	15 (45.5%)	11 (47.8%)	4 (40%)
Chemotherapy	1 (3.0%)	1 (4.3%)	0
Radiation	1 (3.0%)	1 (4.3%)	0
**Place in treatment at baseline, *n* (%)**			
Had begun chemotherapy	25 (76%)	17 (74%)	8 (80%)
Chemotherapy-naïve	8 (24%)	6 (26%)	2 (20%)
**Type of chemotherapy, *n* (%)**			
Doxorubicin Cyclophosphamide (AC) *	11 (33.3%)	7 (30.4%)	4 (40%)
Paclitaxel (with or without Trastuzumab)	7 (21.3%)	4 (17.4%)	3 (30%)
Docetaxel Cyclophosphamide (TC)	4 (12.1%)	2 (8.7%)	2 (20%)
Docetaxel Carboplatin Trastuzumab Pertuzumab (TCHP)	7 (21.2%)	6 (26.1%)	1 (10%)
Other (all non-anthracycline)	4 (12.1%)	4 (17.4%)	0

* Sometimes followed by taxane-based chemotherapy with or without targeted therapy.

**Table 2:** The effects of a Mediterranean Diet (MedDiet) intervention on cancer-related fatigue during chemotherapy treatment (*n* = 33).

Fatigue Measure	Direct-Ionality	Group	Baseline (Mean ± SD)	Week 4 (Mean ± SD)	Effect Size (95% CI)	Week 8 (Mean ± SD)	Effect Size (95% CI)
Functional Assessment of Chronic Illness Therapy-Fatigue (FACIT-F) Total score	Higher is better	Control	115.4 ± 20.5	110.2 ± 26.4	0.31 (−0.44–1.06)	116.9 ± 20.4	0.30 (−0.44–1.05)
		MedDiet	104.5 ± 28.7	107.5 ± 31.9		114.1 ± 28.4	
FACIT-F: Physical well-being	Higher is better	Control	20.6 ± 3.9	19.1 ± 5.7	0.22 (−0.53–0.96)	20.8 ± 3.9	0.18 (−0.56–0.93)
		MedDiet	19.5 ± 6.7	19.3 ± 6.6		20.8 ± 5.9	
FACIT-F: Social well-being	Higher is better	Control	23.8 ± 3.3	24.6 ± 2.2	−0.03 (−0.78–0.71)	23.3 ± 3.7	0.50 (−0.25–1.26)
		MedDiet	22.2 ± 2.8	22.9 ± 3.4		23.2 ± 3.3	
FACIT-F: Emotional well-being	Higher is better	Control	17.8 ± 3.7	17.8 ± 4.5	0.17 (−0.58–0.91)	18.8 ± 2.6	0.09 (−0.65–0.84)
		MedDiet	16.6 ± 4.5	17.3 ± 4.0		18.0 ± 3.8	
FACIT-F: Functional well-being	Higher is better	Control	17.9 ± 5.9	16.8 ± 5.9	0.10 (−0.64–0.85)	18.0 ± 5.6	0.03 (−0.71–0.77)
		MedDiet	16.5 ± 7.1	16.1 ± 7.7		16.8 ± 7.1	
FACIT-F: Fatigue subscale	Higher is better	Control	35.3 ± 10.3	31.9 ± 12.7	0.31 (−0.44–1.06)	36.0 ± 9.8	0.25 (−0.50–0.99)
		MedDiet	31.3 ± 14.4	32.0 ± 14.2		35.3 ± 12.1	
FACIT-F: Trial outcome index (fatigue)	Higher is better	Control	73.8 ± 18.4	67.8 ± 23.0	0.25 (−0.50–0.99)	74.8 ± 18.2	0.19 (−0.55–0.94)
		MedDiet	67.3 ± 26.6	67.3 ± 26.9		73.0 ± 23.7	
FACIT-F: Functional Assessment of Cancer Therapy (FACT)-General	Higher is better	Control	80.1 ± 11.9	78.3 ± 14.8	0.22 (−0.52–0.97)	80.9 ± 11.0	0.26 (−0.49–1.00)
		MedDiet	74.1 ± 15.8	75.6 ± 19		78.7 ± 17.1	
Brief Fatigue Inventory: Global fatigue score	Lower is better	Control	2.9 ± 2.4	3.3 ± 2.6	−0.04 (−0.78–0.70)	2.8 ± 2.2	−0.32 (−1.07–0.43)
		MedDiet	3.4 ± 3.1	3.7 ± 2.7		2.4 ± 2.3	
Brief Fatigue Inventory: Usual fatigue	Lower is better	Control	3.6 ± 2.7	4.0 ± 2.7	0.07 (−0.67–0.82)	2.6 ± 2.0	0.04 (−0.71–0.78)
		MedDiet	3.4 ± 2.8	4.0 ± 3.1		2.5 ± 2.2	
Brief Fatigue Inventory: Fatigue at its worst	Lower is better	Control	5.3 ± 2.9	5.3 ± 3.7	0.15 (−0.59–0.90)	4.2 ± 3.0	0.28 (−0.47–1.02)
		MedDiet	4.2 ± 3.4	4.7 ± 3.2		4.0 ± 3.0	
Symptom inventory: Fatigue	Lower is better	Control	4.3 ± 2.5	5.7 ± 3.6	−0.26 (−1.00–0.49)	4.0 ± 2.7	−0.10 (−0.84–0.65)
		MedDiet	4.8 ± 3.4	5.4 ± 3.2		4.2 ± 3.1	
Symptom inventory: Sleep problems	Lower is better	Control	4.0 ± 3.5	4.1 ± 3	0.03 (−0.71–0.77)	3.5 ± 2.8	0.06 (−0.69–0.80)
		MedDiet	3.5 ± 3.7	3.7 ± 2.9		3.2 ± 2.6	
Symptom inventory: Drowsiness	Lower is better	Control	3.8 ± 2.4	4.1 ± 3.1	−0.10 (−0.85–0.64)	3.7 ± 2.5	−0.14 (−0.88–0.61)
		MedDiet	4.0 ± 3.2	4.0 ± 3.4		3.5 ± 2.8	
Symptom inventory: Interference of symptoms with quality of life	Lower is better	Control	2.6 ± 2.3	2.5 ± 2.6	0.12 (−0.62–0.87)	1.6 ± 1.9	−0.22 (−0.96–0.53)
		MedDiet	4.0 ± 3.5	4.3 ± 3.6		2.3 ± 2.1	

**Table 3:** Associations between mitochondrial measures and cancer-related fatigue incorporating data from baseline and four weeks. The mixed model had a first-order autoregressive repeated structure (AR[1]), fatigue as the dependent variable, participant as a random effect, and age and mitochondrial measure as fixed effects (*n* = 30). For the Functional Assessment of Chronic Illness Therapy-Fatigue (FACIT-F), a higher score indicates less fatigue and a greater quality of life. For the Brief Fatigue Inventory and Symptom Inventory, a higher score indicates higher fatigue.

Fatigue Measure	Basal Respiration (Mean ± SD)	*p*-Value	Maximal Capacity (Mean ± SD)	*p*-Value	Spare Capacity (Mean ± SD)	*p*-Value
Functional Assessment of Chronic Illness Therapy-Fatigue (FACIT-F): Total score	23.67 ± 21.12	0.272	8.80 ± 5.11	0.093	10.00 ± 6.27	0.118
FACIT-F: Physical well-being	7.14 ± 5.35	0.190	1.31 ± 1.25	0.300	1.30 ± 1.49	0.386
FACIT-F: Social well-being	−2.69 ± 2.66	0.321	−0.13 ± 0.61	0.835	−0.01 ± 0.73	0.988
FACIT-F: Emotional well-being	0.80 ± 3.35	0.813	−0.07 ± 0.82	0.930	−0.17 ± 0.99	0.861
FACIT-F: Functional well-being	7.97 ± 3.90	0.057	2.52 ± 0.99	0.019 *	3.13 ± 1.27	0.022 *
Functional Assessment of Cancer Therapy- General (FACT-G)	12.83 ± 14.14	0.375	3.37 ± 2.95	0.262	3.75 ± 3.62	0.307
FACIT-F: Fatigue subscale	20.26 ± 9.63	0.044	5.60 ± 2.33	0.021 *	6.51 ± 2.87	0.029 *
Trial Outcome Index	35.51 ± 18.31	0.062	9.29 ± 4.45	0.044 *	10.60 ± 5.47	0.059
Brief Fatigue Inventory: Total score	−4.49 ± 1.81	0.019 *	−0.87 ± 0.51	0.096	−0.79 ± 0.59	0.185
Brief Fatigue Inventory: Usual fatigue	−5.91 ± 1.99	0.006 *	−1.39 ± 0.53	0.014 *	−1.40 ± 0.67	0.044 *
Brief Fatigue Inventory: Worst fatigue	−4.57 ± 2.58	0.086	−1.11 ± 0.68	0.109	−1.19 ± 0.81	0.149
Symptom Inventory: Fatigue	−2.66 ± 2.38	0.272	−0.89 ± 0.57	0.129	−1.08 ± 0.70	0.129
Symptom Inventory: Sleep problems	−2.39 ± 3.07	0.442	−0.31 ± 0.70	0.666	−0.25 ± 0.83	0.762
Symptom Inventory: Drowsiness	−5.50 ± 2.55	0.037 *	−1.06 ± 0.61	0.091	−1.08 ± 0.74	0.153
Symptom Inventory: How do symptoms interfere with quality of life?	−4.64 ± 2.44	0.067	−1.14 ± 0.59	0.062	−1.27 ± 0.73	0.088

* *p* < 0.05.

## Data Availability

Raw data and statistical coding are available from A.S.K. upon reasonable request.

## Supplementary Materials

The following supporting information can be downloaded at: https://www.mdpi.com/article/10.3390/cancers14174202/s1, Figure S1: Blood-based lipid measures, homocysteine, and fructosamine, as well as body weight, at baseline, week 4, and week 8 for those in the control and Mediterranean Diet groups; Figure S2: Representative raw trace from the Seahorse XFe96 extracellular flux instrument; Table S1: The effect of a Mediterranean Diet (MedDiet) intervention on Mediterranean Diet adherence score and nutrient intake; Table S2: Average of number of servings in each food group per day; Table S3: Baseline associations between Mediterranean Diet adherence and cancer-related fatigue; Table S4: Associations between Mediterranean Diet adherence and cancer-related fatigue over three time points—baseline, week 4, and week 8.

## Appendix A

For mitochondrial assays, T cells were extracted using immunomagnetic negative selection (“The Big Easy” EasySep™ Magnet and EasySep™ Human T cell isolation kit, Stemcell Technologies, Vancouver, Canada) according to the manufacturer’s instructions. Cells were plated at 200,000 cells/well onto a Seahorse 96-well utility plate (Agilent Technologies, Santa Clara, CA, USA) that had been pre-treated with poly-L-lysine for immobilization of T cells (12 technical replicates). Cellular respiration was measured on the Seahorse XFe96 Extracellular Flux Analyzer (Agilent) using the XF Cell Mito Stress test as per the manufacturer’s instructions. We used XF DMEM medium, pH 7.4, with 1 mM pyruvate, 2 mM glutamine, and 10 mM glucose. Metabolic inhibitors were prepared in the media to yield final working concentrations as follows: oligomycin (2.0 µM), carbonilcyanide p-triflouromethoxyphenylhydrazone (FCCP; 2.0 µM), and rotenone (1.0 µM) + antimycin A (1.0 µM; Seahorse XF Cell Mito Stress Test Kit, Agilent). The assay was run using Wave software (v. 2.6.3, Agilent); after calibration and equilibration, three baseline readings were made, and three readings were made after each subsequent injection of a metabolic inhibitor. Cells were then imaged using the Celigo Cell Image Cytometer (Nexcelom Bioscience, Lawrence, MA, USA) to ensure acceptable purity and confluence. Oxygen consumption rate (pmol/min/200,000 cells) was estimated at baseline and after each injection by averaging the three measurements (Wave Software, Agilent). Baseline respiration, maximal respiratory capacity, and spare capacity were calculated using established procedures [64,72].
