# Supplementary material for: The Effects of a Mediterranean Diet Intervention on Cancer-Related Fatigue for Patients Undergoing Chemotherapy: A Pilot Randomized Controlled Trial
Source: Cancers (Basel). 2022 Aug 30;14(17):4202. doi: 10.3390/cancers14174202 (PMC9454611; doi:10.3390/cancers14174202)
Supplement: Supplementary file 1 [file cancers-14-04202-s001.zip › cancers-1854398-supplementary.pdf]

Kleckner, *et al.*, The effects of a Mediterranean Diet intervention on cancer-related fatigue among patients undergoing chemotherapy: A pilot randomized controlled trial

Supplemental Materials

Figure S1: Blood-based lipid measures, homocysteine, and fructosamine, as well as body weight, at baseline, week 4, and week 8 for those in the control and Mediterranean Diet groups

Figure S2: Representative raw trace from the Seahorse XFe96 extracellular flux instrument

Table S1: The effect of a Mediterranean Diet (MedDiet) intervention on Mediterranean Diet adherence score and nutrient intake

Table S2: Average of number of servings in each food group per day

Table S3: Baseline associations between Mediterranean Diet adherence and cancer-related fatigue

Table S4: Associations between Mediterranean Diet adherence and cancer-related fatigue over three time points—baseline, week 4, and week 8.

Figure S1. Blood-based lipid measures, homocysteine, and fructosamine, as well as body weight, at baseline, week 4, and week 8 for those in the control (*n*=10) and Mediterranean Diet (MedDiet) groups (*n*=20 for blood-based markers and *n*=23 for body weight). ES=effect size, which denotes the effect of the intervention from baseline to week 4 or baseline to week 8. **p*<0.05, †*p*<0.10 in an analysis of covariance (ANCOVA), for difference between groups at weeks 4 or 8, controlling for value at baseline. HDL=high-density cholesterol, LDL=low-density cholesterol, NHDLC=non-high-density lipoprotein cholesterol

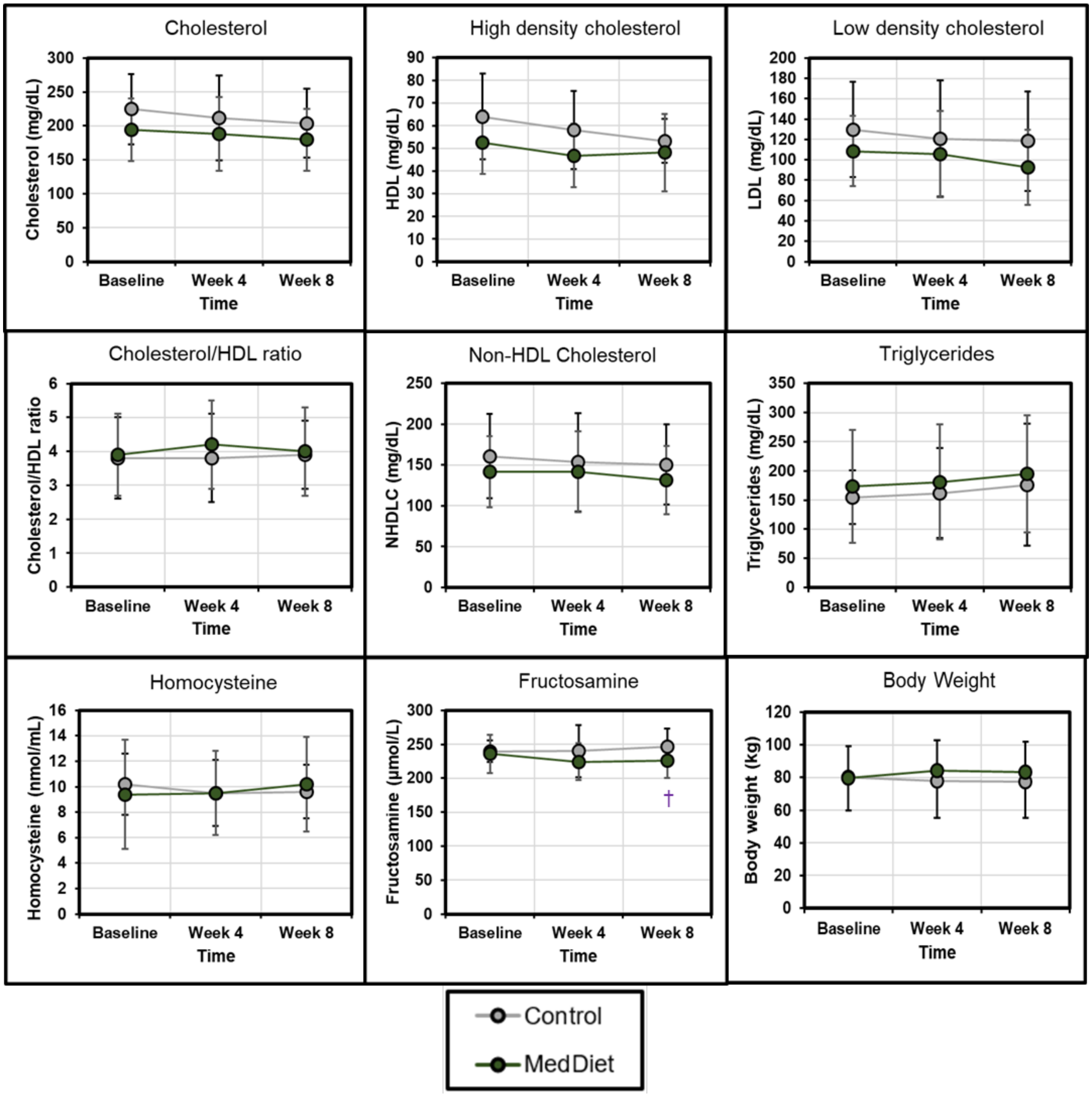

Figure S2. Representative raw trace from the Seahorse XFe96 extracellular flux instrument. Error bars depict standard deviation from 9 technical replicates.

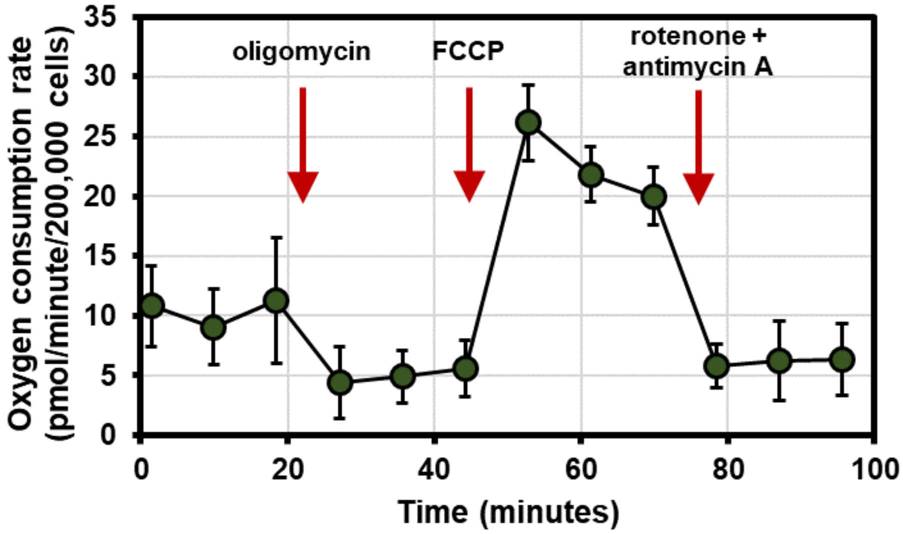

Table S1. The effect of a Mediterranean Diet (MedDiet) intervention on Mediterranean Diet adherence score (*n*=33) and nutrient intake (*n*=25). To assess between-group differences, a mixed model was constructed to estimate outcomes (e.g, MedDiet Score, energy) at 4 or 8 weeks with group, time, and group×time as fixed effects, and baseline value as an independent variable. Average between-group differences were estimated using marginal means estimates.

		**Control**			**MedDiet**				
**Nutrient**	**Re-commended Daily Allowance (RDA)***	**Baseline (mean ± SD)**	**4 weeks (mean ± SD)**	**8 weeks (mean ± SD)**	**Baseline (mean ± SD)**	**4 weeks (mean ± SD)**	**8 weeks (mean ± SD)**	**Average Between-Group Difference in Change from Baseline (MedDiet-Control) ± SE**	***p*-value**
MedDiet score[†]	Not determined	4.7 ± 1.9	5.7 ± 3.0	5.2 ± 3.0	4.0 ± 2.7	6.7 ± 2.8	6.5 ± 2.6	-1.7 ± 1.0	0.080
Energy (kcal)	Not determined	1855 ± 537	1744 ± 531	1418 ± 253	1716 ± 289	1652 ± 360	1701 ± 371	10 ± 156	0.949
Fat (g)	Not determined	77.3 ± 31.2	75.0 ± 27.2	63.4 ± 14.1	73.5 ± 11.4	69.8 ± 18.8	76.1 ± 19.7	-0.4 ± 9.0	0.963
Carbohydrates (g)	130	224.6 ± 57.3	203.6 ± 66.4	158.1 ± 39.7	199.0 ± 63.8	190.6 ± 51.1	192.5 ± 50.9	11.8 ± 18.8	0.539
Protein (g)	56 (males), 46 (females)	71.4 ± 26.9	69.9 ± 13.4	63.9 ± 11.1	73.3 ± 12.3	75.7 ± 20.6	72.2 ± 15.0	-7.4 ± 5.9	0.227
Fiber (g)	38 (males 19-50 y), 30 (males >50 y), 25 (females 19-50 y), 21 (females >50 y)	22.0 ± 3.9	17.2 ± 4.6	20.0 ± 5.8	18.5 ± 7.2	23.5 ± 8.8	22.5 ± 10.1	0.2 ± 5.5	0.977
Saturated fat (g)	Not determined	26.7 ± 13.3	26.2 ± 11.4	18.4 ± 9.5	24.9 ± 8.3	19.9 ± 5.9	21.9 ± 8.0	1.1 ± 3.2	0.734
Monounsaturated fat (g)	Not determined	26.8 ± 10.3	28.4 ± 13.3	24.1 ± 4.8	25.1 ± 5.0	27.4 ± 11.5	30.8 ± 12.7	-4.0 ± 4.8	0.407
Polyunsaturated fat (g)	Not determined	16.8 ± 7.9	18 ± 10.5	17.4 ± 6.4	16.4 ± 3.1	13.7 ± 4.5	17.0 ± 6.7	2.5 ± 2.9	0.404
Cholesterol (g)	Not determined	227.3 ± 140.8	224.5 ± 119.7	206.0 ± 176.3	335.0 ± 125.9	256.4 ± 121.0	310.0 ± 129.0	-55.7 ± 57.4	0.345
Total Vitamin A Activity (International Units) (IU)	900 (males), 700 (females)	11739 ± 8863	6258 ± 5970	8985 ± 5274	8165 ± 5039	12079 ± 10157	9856 ± 7200	-4315 ± 3192	0.180

Vitamin D (calciferol) (mcg)	15 (19-70 y), 20 (>70 y)	29.8 ± 47.8	28.7 ± 55.8	9.8 ± 19.1	23.4 ± 22.3	11.6 ± 19.0	8.1 ± 15.0	7.8 ± 7.6	0.322
Vitamin E (Total Alpha-Tocopherol) (mg)	15	12.4 ± 5.3	11.1 ± 6.8	10.8 ± 4.2	10.5 ± 5.8	11.9 ± 9.1	12.5 ± 10.0	-3.0 ± 3.0	0.317
Vitamin C (ascorbic acid) (mg)	90 (males), 75 (females)	112.7 ± 67.8	106.5 ± 75.9	110.4 ± 68.4	69.6 ± 37	110.1 ± 68.3	106.3 ± 63.2	-1564.9 ± 1711.4	0.366
Thiamin (vitamin B1) (mg)	1.2 (males), 1.1 (females)	1.6 ± 0.5	1.4 ± 0.4	1.4 ± 0.3	1.5 ± 0.6	1.4 ± 0.6	1.3 ± 0.6	0.0 ± 0.2	0.994
Riboflavin (vitamin B2) (mg)	1.3 (males), 1.1 (females)	2.1 ± 0.7	1.9 ± 0.9	1.6 ± 0.4	1.9 ± 0.7	1.7 ± 0.7	1.7 ± 0.7	-0.1 ± 0.3	0.805
Niacin (vitamin B3) (mg)	16 (males), 14 (females)	21.3 ± 8.2	19.7 ± 6.5	19.1 ± 5	22.6 ± 7	22.4 ± 8.1	21.3 ± 8.4	-2.1 ± 2.8	0.463
Pantothenic Acid (mg)	Not determined	6.4 ± 2.9	5.2 ± 2.7	4.9 ± 1.3	6.4 ± 3.3	5.5 ± 3.2	5.5 ± 3.6	-0.5 ± 1.2	0.691
Vitamin B-6 (pyridoxine, pyridoxyl, & pyridoxamine) (mg)	1.3 (19-50 y), 1.7 (males >50 y), 1.5 (females >50 y)	1.9 ± 0.9	1.6 ± 0.6	1.5 ± 0.5	2.0 ± 0.9	2.1 ± 0.9	2.0 ± 1.0	-0.4 ± 0.4	0.236
Total Folate (mcg)	400	360.0 ± 140.7	302.6 ± 118.6	353.7 ± 87.7	370.5 ± 157.7	405.3 ± 191.5	351.6 ± 242	-38.5 ± 75.7	0.617
Vitamin B-12 (cobalamin) (mcg)	2.4	4.0 ± 2.5	6.0 ± 5.9	3.5 ± 2.5	5.7 ± 3.1	3.9 ± 2	4.7 ± 4.2	1.1 ± 1.5	0.468
Calcium (mg)	1000 (19-50 y, males 51-70 y), 1200 (females 51-70 y, >70y)	977.0 ± 399.9	889.8 ± 471.1	658.2 ± 178.4	921.1 ± 478.1	851.7 ± 500.7	750.5 ± 383.6	-56.3 ± 164.6	0.736
Phosphorus (mg)	700	1193.0 ± 395.6	1128.0 ± 394.8	1001.6 ± 251.7	1151.5 ± 260.7	1147.4 ± 288.7	1141.9 ± 348.9	-101.9 ± 112.4	0.377
Magnesium (mg)	400 (males 19-30), 420 (males >30 y), 310 (females 19-30 y), 320 (females >30 y)	300.0 ± 65.1	256.6 ± 73.3	261.5 ± 106	252.2 ± 61.8	330.1 ± 121.7	304.5 ± 144.3	-118.0 ± 43.8	0.015
Iron (mg)	8 (males, females >50 y), 18 (females 19-50 y)	12.5 ± 5.4	11.3 ± 3.4	10.4 ± 3	14.7 ± 6.6	14.1 ± 6.3	12.9 ± 7.8	-1.1 ± 2.2	0.641
Zinc (mg)	11 (males), 8 (females)	10.1 ± 5.1	8.1 ± 2.5	7.0 ± 2.2	11.0 ± 4.5	10.3 ± 5.3	10.2 ± 5.6	-2.6 ± 1.9	0.187
Copper (mg)	900	1.1 ± 0.3	0.9 ± 0.2	0.9 ± 0.3	1.2 ± 0.4	1.4 ± 0.7	1.4 ± 0.9	-0.5 ± 0.3	0.095
Selenium (mcg)	55	101.8 ± 36.6	103.9 ± 19.5	97.1 ± 30.0	116.4 ± 23.6	101.1 ± 34.0	124.6 ± 39.0	-8.5 ± 15.0	0.577

Sodium (mg)	1500	2885.4 ± 1477.8	2841.8 ± 1290.4	2474.3 ± 1215.1	2848.5 ± 847.7	2502.4 ± 590.2	2441.7 ± 675.1	242.1 ± 294.0	0.421
Potassium (mg)	3400 (males), 2600 (females)	2420.8 ± 736.8	2234.0 ± 653.5	2195.7 ± 661.1	2283.3 ± 611.2	2650.9 ± 1024.9	2614.3 ± 1170.5	-483.1 ± 401.9	0.230

*RDA: Recommended Daily Allowances from the National Academy of Medicine (NAM) of the National Academies (United States). These represent the estimated average daily level of intake sufficient to meet the nutrient requirements of nearly all (97%-98%) healthy people.

†MedDiet score: Adherence to the Mediterranean Diet using the 14-item Mediterranean Diet Assessment Tool [34], a higher score indicates higher adherence

Table S2. Average of number of servings in each food group per day, as measured using 3-day food records. To assess between-group differences, a mixed model was constructed to estimate the number of servings at 4 or 8 weeks with group, time, and group×time as fixed effects, and the number of servings at baseline as an independent covariate. Average between-group differences were estimated using marginal means estimates.

	**Control (*n*=7-8)**			**MedDiet (*n*=16)**				
**Food group***	**Baseline (mean± SD)**	**Week 4 (mean± SD)**	**Week 8 (mean± SD)**	**Baseline (mean± SD)**	**Week 4 (mean± SD)**	**Week 8 (mean± SD)**	**Average Between-Group Difference in Change from Baseline (MedDiet-Control) ± SE**	***p*-value**
Fruit	2.1 ± 1.1	1.6 ± 1.4	1.7 ± 1.6	1.9 ± 1.8	1.2 ± 1.2	1.4 ± 1.2	-0.37 ± 0.56	0.524
Vegetables	3.0 ± 2.4	2.3 ± 2.5	3.4 ± 2.1	2.2 ± 1.1	3.5 ± 1.8	3.6 ± 2.6	0.92 ± 0.75	0.232
Potatoes and other starchy vegetables	0.2 ± 0.2	0.6 ± 0.6	0.3 ± 0.5	0.6 ± 0.7	0.5 ± 0.4	0.8 ± 0.8	0.16 ± 0.23	0.482
Beans and legumes	0.3 ± 0.8	0.1 ± 0.2	0.1 ± 0.3	0.2 ± 0.3	0.4 ± 0.4	0.4 ± 0.5	0.29 ± 0.15	0.076
Whole grains	0.7 ± 0.8	1.0 ± 1.3	1.0 ± 2.0	0.6 ± 0.7	1.8 ± 1.3	1.8 ± 1.3	1.08 ± 0.47	0.034[†]
Grains, some whole grain	0.8 ± 1.7	0.4 ± 0.5	0.8 ± 1.6	0.6 ± 0.6	0.4 ± 0.5	0.5 ± 0.8	-0.13 ± 0.26	0.622
Refined grains	3.3 ± 1.4	3.5 ± 2.2	2.8 ± 2.4	3.8 ± 1.7	2.4 ± 1.9	2.2 ± 1.6	-1.40 ± 0.77	0.084
Meat and poultry	3.5 ± 3.0	3.1 ± 1.9	2.5 ± 1.4	3.1 ± 1.1	3.0 ± 1.4	2.4 ± 1.9	0.25 ± 0.49	0.606
Fish	0.1 ± 0.2	0.7 ± 1.1	0.7 ± 1.1	1.1 ± 1.0	0.9 ± 0.8	1.5 ± 1.3	0.54 ± 0.46	0.253
Eggs	0.3 ± 0.5	0.4 ± 0.3	0.5 ± 0.8	0.8 ± 0.7	0.7 ± 0.6	1.0 ± 0.7	0.37 ± 0.25	0.154
Nuts	0.9 ± 1.3	0.3 ± 0.5	0.7 ± 1.0	0.8 ± 0.8	0.9 ± 0.7	1.3 ± 1.6	0.63 ± 0.38	0.114
Meat alternatives	0 ± 0	0.1 ± 0.3	0.1 ± 0.3	0 ± 0	0.3 ± 0.5	0.1 ± 0.4	0.00 ± 0.10	0.966
Dairy	3.9 ± 2.6	2.7 ± 2.5	2.7 ± 1.9	3.2 ± 1.5	2.1 ± 1.5	2.4 ± 1.4	-0.10 ± 0.74	0.889
Oil	1.5 ± 1.2	1.7 ± 1.5	2.3 ± 1.5	1.6 ± 0.7	3.1 ± 3.1	3.1 ± 2.8	1.02 ± 1.21	0.410
Butter and shortening	2.1 ± 2.9	1.7 ± 2.1	0.8 ± 1.5	1.2 ± 1.5	0.5 ± 0.7	1.6 ± 2.7	0.23 ± 0.60	0.708
Sweets	0.8 ± 0.5	0.8 ± 1.0	0.9 ± 1.2	1.0 ± 1.1	1.0 ± 1.5	1.0 ± 1.2	-0.01 ± 0.39	0.975
Sweetened beverages	1.6 ± 1.9	0.5 ± 0.7	0.5 ± 0.9	0.5 ± 0.6	0.2 ± 0.6	0.4 ± 0.7	-0.12 ± 0.32	0.705

*NDSR codes: Fruits includes fruit, fruit juice, avocado, fried fruits, and fruit-based savory snacks (FRU0100-FRU0700). Vegetables includes dark green vegetables (VEG0100), deep yellow vegetables (VEG0200), tomatoes (VEG0300), other vegetables (VEG0600), fried vegetables (VEG0900), vegetable juice (VEG0500), and vegetable-based savory snacks (FMC0100). It does not include white or fried potatoes, other starchy vegetables, or legumes. Potatoes and other starchy vegetables includes white potatoes (VEG0400), fried potatoes (VEG0800), and other starchy vegetables (VEG0450). Beans and legumes includes only legumes (cooked dried beans; VEG0700). Whole grains includes whole grains, flour, and dry mixes; loaf-type bread and plain rolls; other breads; crackers; pasta; ready-to-eat cereals; cakes, cookies, pies, pastries, Danishes, doughnuts, and cobblers;

snack bars; snack chips, and popcorn (all codes beginning GRW). Grains, some whole grain includes the same categories as whole grain except popcorn, but with some of the grains whole grain (all codes beginning GRS). Refined grains includes the same categories as whole grain except popcorn, plus baby food grain mixtures (all codes beginning GRR). Meat and poultry include all meat and poultry categories except the five fish categories, including beef, lamb, pork, poultry, cold cuts, sausage, meat-based savory snacks, and others (19 total). Fish includes fish and shellfish (MFF0100, MFL0100, MFF0200, MSL0100, MSF0100). Eggs includes eggs (MOF0300) and egg substitutes (MOF0400). Nuts includes nuts and seeds (MOF0500) and nut and seed butters (MOF0600). Meat alternatives includes soy-, pea protein-, and other protein-based formulations used to mimic chicken nuggets, ground beef, etc. (MOF0700). Dairy includes milk, cheese, yogurt, frozen desserts, cream, and margarine (40 categories). Oil includes oil (FOF0100) and salad dressings (FDF0100, FDR0100). Sweets includes sugar, syrup, honey, jelly, chocolate, candy, frosting, etc. (SWT0100-SWT0500, SWT0700, SWT0800, MSC1200). Sweetened beverages includes sweetened and artificially sweetened soft drinks, fruit drinks, tea, coffee, and water (all categories starting BVS and BVA), but not any unsweetened beverages.

[†] $p<0.05$

Table S3. Baseline associations between Mediterranean Diet adherence and cancer-related fatigue. Linear models are adjusted for age (*n*=33). For the Functional Assessment of Chronic Illness Therapy-Fatigue (FACIT-F), a higher score indicates less fatigue and a greater quality of life. For the Brief Fatigue Inventory and Symptom Inventory, a higher score indicates higher fatigue. *$p<0.05$

Measure	Estimate ± SE	*p*-value
**FACIT-F: Total score**	2.92 ± 1.78	0.111
**FACIT-F: Physical well-being**	0.54 ± 0.38	0.163
**FACIT-F: Social well-being**	0.02 ± 0.22	0.926
**FACIT-F: Emotional well-being**	0.01 ± 0.31	0.984
**FACIT-F: Functional well-being**	0.46 ± 0.49	0.355
**FACIT-F: Fatigue subscale**	1.81 ± 0.87	0.046*
**FACIT-F: Trial Outcome Index**	2.82 ± 1.63	0.095
**FACIT-F: Functional Assessment of Cancer Therapy- General (FACT-G)**	1.07 ± 1.03	0.311
**Brief Fatigue Inventory: Total score**	-0.38 ± 0.17	0.031*
**Brief Fatigue Inventory: Usual fatigue**	-0.39 ± 0.18	0.040*
**Brief Fatigue Inventory: Worst fatigue**	-0.59 ± 0.21	0.009*
**Symptom Inventory: Fatigue**	-0.34 ± 0.22	0.130
**Symptom Inventory: Sleep problems**	-0.12 ± 0.26	0.654
**Symptom Inventory: Drowsiness**	-0.21 ± 0.21	0.310
**Symptom Inventory: How do symptoms interfere with quality of life**	-0.54 ± 0.22	0.017*

Table S4. Associations between Mediterranean Diet adherence and cancer-related fatigue over three time points—baseline, week 4, and week 8. The mixed model has a first-order autoregressive repeated structure (AR[1]) with random effect for participant and age and Mediterranean Diet adherence score (MedDiet) as independent variables (*n*=33). For the Functional Assessment of Chronic Illness Therapy-Fatigue (FACIT-F), a higher score indicates less fatigue and a greater quality of life. For the Brief Fatigue Inventory and Symptom Inventory, a higher score indicates higher fatigue. **p*<0.05

Measure	Estimate ± SE	*p*-value
**FACIT-F: Total score**	2.360 ± 0.842	0.007*
**FACIT-F: Physical well-being**	0.545 ± 0.200	0.008*
**FACIT-F: Social well-being**	0.088 ± 0.109	0.419
**FACIT-F: Emotional well-being**	0.074 ± 0.123	0.545
**FACIT-F: Functional well-being**	0.318 ± 0.170	0.066
**FACIT-F: Fatigue subscale**	1.440 ± 0.439	0.002*
**FACIT-F: Trial Outcome Index**	2.272 ± 0.762	0.004*
**FACIT-F: Functional Assessment of Cancer Therapy- General (FACT-G)**	1.009 ± 0.431	0.022*
**Brief Fatigue Inventory: Total score**	-0.303 ± 0.080	<0.001*
**Brief Fatigue Inventory: Usual fatigue**	-0.300 ± 0.094	0.002*
**Brief Fatigue Inventory: Worst fatigue**	-0.382 ± 0.109	0.001*
**Symptom Inventory: Fatigue**	-0.223 ± 0.118	0.063
**Symptom Inventory: Sleep problems**	-0.111 ± 0.103	0.287
**Symptom Inventory: Drowsiness**	-0.188 ± 0.110	0.091
**Symptom Inventory: How do symptoms interfere with quality of life**	-0.313 ± 0.109	0.005*
